# Deconvolution-Based CT and MR Brain Perfusion Measurement: Theoretical Model Revisited and Practical Implementation Details

**DOI:** 10.1155/2011/467563

**Published:** 2011-08-28

**Authors:** Andreas Fieselmann, Markus Kowarschik, Arundhuti Ganguly, Joachim Hornegger, Rebecca Fahrig

**Affiliations:** ^1^Pattern Recognition Lab, Department of Computer Science, Friedrich-Alexander University of Erlangen-Nuremberg, Martensstraße 3, 91058 Erlangen, Germany; ^2^Erlangen Graduate School in Advanced Optical Technologies (SAOT), Friedrich-Alexander University of Erlangen-Nuremberg, 91052 Erlangen, Germany; ^3^Siemens AG, Healthcare Sector, Angiography & Interventional X-Ray Systems, Siemensstraße 1, 91301 Forchheim, Germany; ^4^Department of Radiology, Lucas MRS Center, Stanford University, 1201 Welch Road, Palo Alto, CA 94305, USA

## Abstract

Deconvolution-based analysis of CT and MR brain perfusion data is widely used in clinical practice and it is still a topic of ongoing research activities. In this paper, we present a comprehensive derivation and explanation of the underlying physiological model for intravascular tracer systems. We also discuss practical details that are needed to properly implement algorithms for perfusion analysis. Our description of the practical computer implementation is focused on the most frequently employed algebraic deconvolution methods based on the singular value decomposition. In particular, we further discuss the need for regularization in order to obtain physiologically reasonable results. We include an overview of relevant preprocessing steps and provide numerous references to the literature. We cover both CT and MR brain perfusion imaging in this paper because they share many common aspects. The combination of both the theoretical as well as the practical aspects of perfusion analysis explicitly emphasizes the simplifications to the underlying physiological model that are necessary in order to apply it to measured data acquired with current CT and MR scanners.

## 1. Introduction

Tissue perfusion measurement from iodinated contrast agent enhancement on CT scans was first proposed by Axel in 1980 [1]; this was based on earlier developments by Meier and Zierler [2] for measuring blood flow and blood volume. At that time, the CT-based measurements were strictly limited to research because of the low speeds and narrow coverage of the existing CT scanners. However, the introduction of perfusion CT (PCT) helped expand the utility of CT significantly since it could now provide capillary level hemodynamic information. Within about a decade, perfusion imaging techniques were also adopted in MR [3–5].

With the advent of helical scanners and faster rotating gantries (0.33–0.5 s/rotation) in conjunction with multidetector geometries which provide larger coverage, PCT has now become part of the routine screening for many diseases.

Given the existing developments in perfusion imaging, the purpose of this paper is to focus on a detailed derivation of the theoretical model for deconvolution-based perfusion measurement. While the main equation of this model is well known, its derivation is spread over several publications.

We therefore first present a summary of the derivation, with the aim of fully explaining the parameters and the underlying assumptions that are made. Based on the main equation of the theoretical model, we also present a guideline for the algorithmic implementation of the deconvolution-based perfusion measurement. We discuss robust numerical deconvolution and discuss topics related to data pre-processing, providing references to the literature for each of the special topics. The overall aim of this paper is to provide an understanding of the underlying assumptions of the theoretical model and to show how the (simplified) model can be robustly implemented for clinical image analysis.

## 2. Clinical Applications of Perfusion Imaging

 Perfusion imaging is most widely used in acute stroke and oncology [6]. When used in diagnosis of stroke, the purpose of perfusion imaging is to identify the extent of affected tissue and to delineate the ischemic tissue that can be reperfused. In oncology, perfusion imaging helps to identify angiogenetic tumors that alter the local tissue perfusion due to generation of neovasculature. Perfusion measurements are increasingly being used for assessment, staging, and monitoring posttherapy [6, 7].

**Figure 1:**
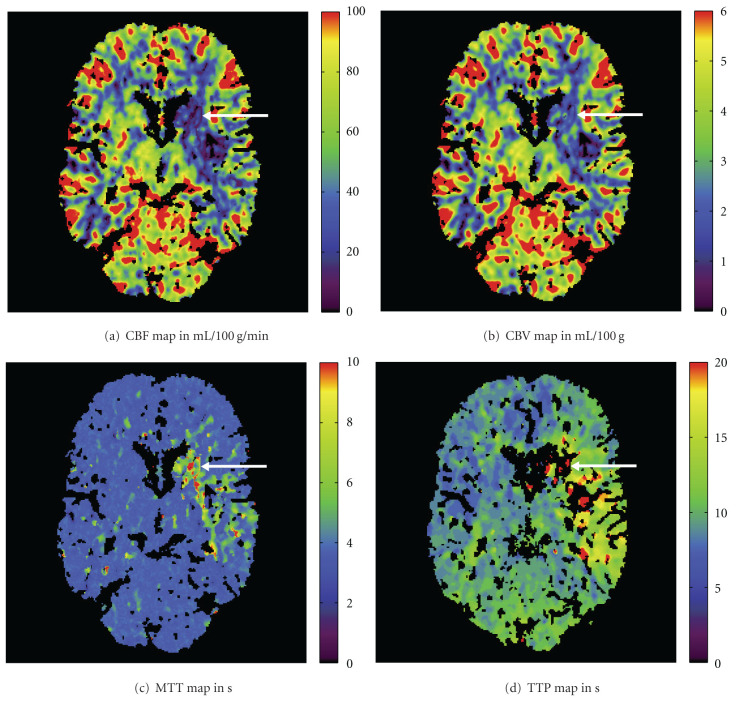
CT perfusion parameter maps of cerebral blood flow (CBF), cerebral blood volume (CBV), mean transit time (MTT), and time-to-peak (TTP). The ischemic stroke lesion is marked with arrows.

Figure 1 shows common parameter maps based on a brain perfusion CT exam (Somatom Definition AS+, Siemens AG, Healthcare Sector, Forchheim, Germany) of a 69-year-old male stroke patient. The patient presented to the hospital with an acute high-grade hemiparesis on the right side. A CT angiography scan indicated an occlusion of the left middle cerebral artery. The time-to-peak (TTP) image shows a large lesion that illustrates the maximum affected tissue. In addition, the cerebral blood flow (CBF), cerebral blood volume (CBV), and mean transit time (MTT) images exhibit perfusion deficits in a smaller brain territory. In general, these perfusion CT maps are interpreted appropriately in order to guide the recanalization procedure of the occluded vessel.

Blood flow is critical to the functionality of any organ since it provides the essential nutrients and oxygen. In case of flow disruption, the body autoregulates the flow and pressure either by altering blood flow or volume or both. In the brain, there are some fairly well-defined thresholds for the cerebral blood flow in normal, reversibly damaged, and necrotic tissue. The normal value for the cerebral blood flow is between 50 and 60 mL/100 g/min for grey matter [8]. The average value decreases with age and is about 2 to 3 times lower in white matter compared to grey matter [9]. Any reduction in normal perfusion pressure results in vasodilation and hence an increase in blood volume and transit times. As the perfusion pressure falls lower, compensatory vasodilation is unable to offset the deficit. When this value falls below 20 mL/100 g/min (for grey matter), synaptic transmission ceases to function. When the flow is below 8–10 mL/100 g/min, the cell membrane pumps fail, causing irreversible damage to the cells [6].

### 2.1. Perfusion CT

 In the acute stroke setting, conventional CT has been the imaging modality of choice for ruling out intracerebral hemorrhages (ICH). However, overall the sensitivity of CT for stroke detection is 60–65% [10, 11]. For ischemic stroke, which represents about 85% of all stroke cases, the inclusion of PCT along with CT angiography (CTA) can identify the subtle abnormalities in the cerebral tissue that can be missed on the noncontrast agent-enhanced scans. Most commonly, the perfusion scan consists of imaging one or two slices at the level of the basal ganglia. This allows inclusion of the branches of the carotid artery that are typically thrombosed. After approximately 7–10 s following an intravenous injection of iodinated contrast agent, continuous scanning is performed for about 50 s. Table toggling techniques are sometimes used to increase the coverage. More recent wide detector scanners allow whole brain coverage in each scan. The temporal scans are reconstructed and one of several approaches can be used to calculate the perfusion parameters.

In animal studies, the product of CT cerebral blood volume (CBV) and flow (CBF) from CT measurements was found to have sensitivity of 90.6% and specificity of 93.3% (compared with histological measurements) for discerning ischemic and oligemic tissue [12]. One study that compared stroke diagnosis using CT perfusion plus angiography, against MRI, found good correlation and no significant prognosis differences [13]. Typical PCT scans add approximately 5 or less additional minutes to the scan time with around a 50 mL bolus of additional iodinated contrast agent. With regards to the X-ray dose in PCT, depending on the parameters, the effective dose is estimated to be between 1.2 mSv [6] and 3.4 mSv [14]. This is in the same range as the effective dose of a standard cerebral CT, which is reported to deliver about 2.5 mSv to the patient [14].

The gold standard for perfusion CT has been imaging with stable xenon as the contrast agent [7, 15]. This method involves inhalation of a mixture of stable xenon gas and oxygen followed by CT scanning. Because of the high atomic number of xenon, it serves as a radio-opaque contrast agent as it diffuses into the blood and neurons in a well-balanced manner. It has been proven to be accurate in quantifying perfusion by comparing the results with those obtained using radio-labeled microspheres.

### 2.2. Perfusion MR

Perfusion imaging in MR can be performed with or without contrast agent [16]. Noncontrast agent-enhanced perfusion imaging usually uses spin labeling of blood entering the imaging volume. This method is less commonly used because of the increased sensitivity to motion and related artifacts and low signal in case of slow flow. Gadolinium-based tracers such as Gd-DTPA are more commonly used for measuring perfusion derived from changes in the local susceptibility. Both spin echo (SE) and gradient echo (GRE) sequences have been applied successfully in perfusion MR. GRE sequences are most frequently used because they provide a better contrast-to-noise ratio for imaging of the contrast agent compared to SE sequences [17–19]. However, GRE sequences have the disadvantage of disproportionately weighting the contribution of the contrast agent in relatively large vessels, whereas SE sequences provide a more accurate assessment of blood flow through vessels of all sizes [19]. After the 7–10 s interval that the gadolinium contrast agent takes to reach the brain following the intravenous injection, the signal in the cerebral tissue dips. The signal changes are most significant over about 15 s during which the change in *T*2* or equivalently the change in the associated relaxation rate *R*2* is monitored. Note that this also requires that the contrast agent is intravascular. Rapid imaging (interval less than 2 s) is required for accurate measurement of perfusion parameters. Typically echo-planar imaging (EPI) sequences are used for this purpose.

## 3. Theoretical Model

The aim of this section is to provide a compact outline of both some elementary as well as practically relevant theory of perfusion estimation based on previous work. In particular, we will introduce a theoretical physiological model of tissue perfusion for intravascular tracer systems and present the derivation of a deconvolution-based mathematical approach for the estimation of diagnostically important perfusion parameters. In addition, we will briefly describe alternative methods that do not require deconvolution.

### 3.1. Model of Microcirculation at the Tissue Level

**Figure 2:**
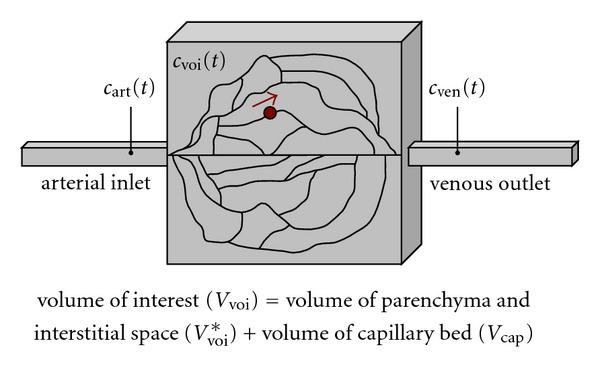
Physiological model of the tissue perfusion. A blood cell can take several paths through the capillary bed. The variables are defined in Table 1.

For computing the tissue perfusion, we assume a physiological model of the blood supply to the tissue.  Figure 2 shows this model that consists of a volume of interest *V*_voi_ covering the organ-specific parenchyma, the interstitial space, and the capillary bed. The volumes of the parenchyma and the interstitial space are denoted by *V*_voi_*, while the volume of the capillary bed is referred to as *V*_cap_. The entire volume of interest *V*_voi_ = *V*_voi_* + *V*_cap_ shall be supplied with blood by a single arterial inlet and correspondingly drained by a single venous outlet. In general, it may have a different shape than the cuboid shown in Figure 2. A blood cell can take various paths through the capillary bed. The transit time *t* it needs to pass through the capillary bed depends on the chosen path. We assume a stationary probability density distribution *h*(*t*) of transit times.

Once a contrast agent bolus has been injected, it enters the volume *V*_voi_ under consideration via the arterial inlet and is then diluted into the capillary bed. The local contrast agent concentrations *c*_art_(*t*) and *c*_ven_(*t*) are measured directly adjacent to the capillary bed on the arterial and venous sides, respectively. Furthermore, the average contrast agent concentration *c*_voi_(*t*) within the volume of interest can also be measured. In perfusion CT, an iodinated contrast agent is used whereas, in perfusion MR, the measured signal difference is created by a paramagnetic contrast agent based on gadolinium (Gd) (see Section 2.2). The contrast agent concentration is defined as mass of iodinated contrast agent per volume (unit: g/mL) or amount of Gd-based contrast agent per volume (unit: mol/mL), respectively [20]. For the following analysis, we assume the contrast agent concentration to be measured as mass per volume, which can easily be related to amount per volume.

**Figure 3:**
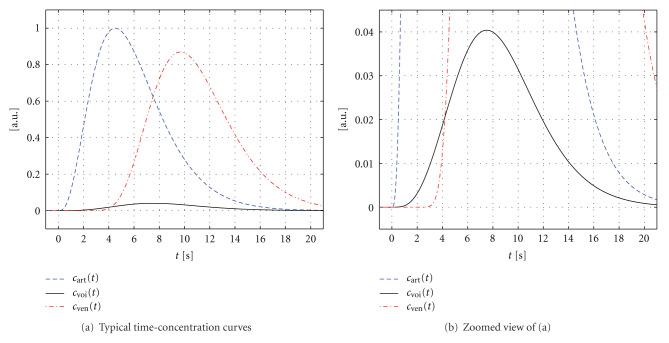
Examples of the time-concentration curves *c*_art_(*t*), *c*_voi_(*t*), and c_ven_(*t*) given in arbitrary units (a.u.). (b) Represents a zoomed view of (a) with a rescaled ordinate.

Figure 3 illustrates typical time-concentration curves *c*_art_(*t*), *c*_voi_(*t*), and *c*_ven_(*t*) that may be measured in brain tissue, for example. For the sake of simplicity, the maximum contrast agent concentration has been normalized to 1. Note that the (average) enhancement within the volume of interest is commonly more than an order of magnitude below the enhancements of the feeding artery and the draining vein.

An additional important assumption is that the contrast agent remains in the intravascular space. For our case of cerebral perfusion, it should therefore not cross the blood-brain barrier (BBB). As a consequence, this means that all contrast agents entering from the arterial inlet will eventually leave the volume of interest at the venous outlet. A breakdown of the BBB may occur in tumor patients, in stroke patients, and in patients that suffer from inflammations or infections, for example. In these cases, the methods presented in this paper may lead to inaccurate perfusion estimates and particularly to an overestimation of the blood volume [21, 22]. Note that there exist other modelling approaches which do not assume that the contrast agent remains in the intravascular space. These models can be used for measuring tumor perfusion, for example [6, 20, 23].

Finally, we suppose that the contrast agent mixes perfectly with the blood and that the physical properties of the blood (its flow behavior, in particular) are not influenced by the contrast agent.

As we will see, only knowledge of the functions *c*_art_(*t*) and *c*_voi_(*t*) is needed to compute the blood flow within the volume under consideration. In practice, the function *c*_art_(*t*)—also known as the arterial input function (AIF)—is not measured directly at the respective volume of interest, but in a larger feeding artery in order to achieve a reasonable signal-to-noise ratio (SNR) (see Section 4.1).

As a first diagnostically relevant perfusion parameter, the mean transit time (MTT) of the volume under consideration is defined as the first moment of the probability density function *h*(*t*) of the transit times, that is,

(1) $$\begin{array}{c} \text{MTT}=\overset{\infty }{\underset{0}{\int }}\tau h\left(\tau \right)\text{d}\tau . \end{array}$$

Furthermore, the residue (or residual) function *r*(*t*)—compare [24]—represents an intermediate quantity of interest and is defined as

(2) $$\begin{array}{c} r\left(t\right)=\{\begin{array}{cc} 1-\overset{t}{\underset{0}{\int }}h\left(\tau \right)\text{d}\tau , & \text{for}\;t\geq 0,\\ 0, & \text{for}\;t<0. \end{array} \end{array}$$

**Figure 4:**
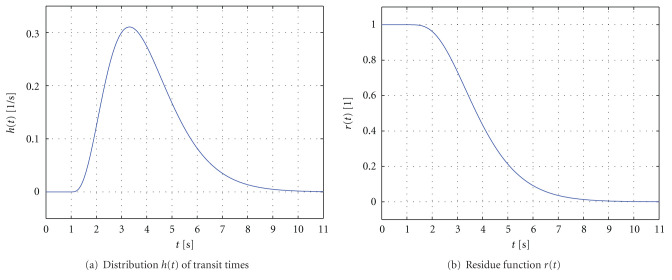
Examples of the distribution function *h*(*t*) of transit times (the mean transit time is 4 s) and the corresponding residue function *r*(*t*).

The (dimensionless) residue function thus quantifies the relative amount of contrast agent that is still inside the volume *V*_voi_ of interest at time *t* after an (idealized) delta-shaped contrast agent bolus has entered the volume at the arterial inlet at time *t* = 0; that is, *c*_art_(*t*) = *δ*(*t*). Due to the various transit times within the capillary bed, the contrast agent will not leave the volume instantaneously, but gradually over time. In particular, this means that the residue function decreases continuously from *r*(0) = 1 to 0.  Figure 4 shows typical examples of a distribution function *h*(*t*) of transit times as well as the corresponding residue function *r*(*t*). In this example, the function *h*(*t*) is modeled by a gamma distribution [25].

### 3.2. Derivation of the Indicator-Dilution Theory

Using the parameters defined in Table 1, the accumulated masses of contrast agent that have entered and left the volume of interest during the time interval [0, *t*], denoted as *m*_*c*,voi,in_(*t*) and *m*_*c*,voi,out_(*t*), respectively, can be expressed as

(3) $$\begin{array}{c} m_{c,\text{voi},\text{in}}\left(t\right)=F\overset{t}{\underset{0}{\int }}c_{\text{art}}\left(\tau \right)\text{d}\tau ,\\ m_{c,\text{voi},\text{out}}\left(t\right)=F\overset{t}{\underset{0}{\int }}c_{\text{ven}}\left(\tau \right)\text{d}\tau . \end{array}$$

The volume flow *F* is assumed to be constant over time. The contrast agent concentrations *c*_art_(*t*) and *c*_ven_(*t*) at the arterial inlet and the venous outlet, respectively, are time-dependent functions which we assume to be 0 for *t* < 0. These functions primarily depend on the parameters of the contrast agent injection and the patient's cardiac cycle.

We can compute the mass *m*_*c*,voi_(*t*) of a contrast agent within the volume of interest at time *t* using the principle of conservation of mass as

(4) $$\begin{array}{cc} m_{c,\text{voi}}\left(t\right) & =m_{c,\text{voi},\text{in}}\left(t\right)-m_{c,\text{voi},\text{out}}\left(t\right)\\ & =F\overset{t}{\underset{0}{\int }}\left(c_{\text{art}}\left(\tau \right)-c_{\text{ven}}\left(\tau \right)\right)\text{d}\tau . \end{array}$$

The contrast agent concentration *c*_ven_(*t*) at the venous outlet can be computed from the contrast agent concentration *c*_art_(*t*) at the arterial inlet by convolving it with the probability density function *h*(*t*). We therefore obtain

(5) $$\begin{array}{c} c_{\text{ven}}\left(t\right)=\overset{+\infty }{\underset{-\infty }{\int }}c_{\text{art}}\left(\xi \right)h\left(t-\xi \right)\text{d}\xi . \end{array}$$

Note that throughout this paper, all integrals with infinite integration endpoints shall be interpreted as the limit of the integral when the respective endpoint approaches ±*∞*. Using (5), we can rewrite (4), by applying the delta function *δ*(*t*), as

(6) $$\begin{array}{cc} & m_{c,\text{voi}}\left(t\right)\\ & =F\overset{t}{\underset{0}{\int }}\left(\overset{+\infty }{\underset{-\infty }{\int }}c_{\text{art}}\left(\xi \right)\delta \left(\tau -\xi \right)\text{d}\xi -\overset{+\infty }{\underset{-\infty }{\int }}c_{\text{art}}\left(\xi \right)h\left(\tau -\xi \right)\text{d}\xi \right)\text{d}\tau . \end{array}$$

Changing the order of integration and rearranging this equation leads to

(7) $$\begin{array}{c} m_{c,\text{voi}}\left(t\right)=F\overset{+\infty }{\underset{-\infty }{\int }}c_{\text{art}}\left(\xi \right)\left(\overset{t}{\underset{0}{\int }}\left(\delta \left(\tau -\xi \right)-h\left(\tau -\xi \right)\right)\text{d}\tau \right)\text{d}\xi . \end{array}$$

By applying the substitution *τ*′ = *τ* − *ξ*, recalling that, for *t* ≥ 0, we have

(8) $$\begin{array}{c} r\left(t\right)=1-\overset{\text{t}}{\underset{0}{\int }}h\left(\tau \right)\text{d}\tau =\overset{t}{\underset{0}{\int }}\left(\delta \left(\tau \right)-h\left(\tau \right)\right)\text{d}\tau , \end{array}$$

and considering that *h*(*t*) = 0 for *t* < 0, we obtain

(9) $$\begin{array}{cc} & \overset{t}{\underset{0}{\int }}\left(\delta \left(\tau -\xi \right)-h\left(\tau -\xi \right)\right)\text{d}\tau \\ & \;\;=\overset{t-\xi }{\underset{-\xi }{\int }}\left(\delta \left(\tau^{\prime }\right)-h\left(\tau^{\prime }\right)\right)\text{d}\tau^{\prime }=r\left(t-\xi \right). \end{array}$$

Equation (7) thus eventually reads

(10) $$\begin{array}{c} m_{c,\text{voi}}\left(t\right)=F\overset{+\infty }{\underset{-\infty }{\int }}c_{\text{art}}\left(\xi \right)r\left(t-\xi \right)\text{d}\xi . \end{array}$$

We introduce the cerebral blood flow (CBF) as the blood volume flow normalized by the mass of the volume *V*_voi_,

(11) $$\begin{array}{c} \text{CBF}=\frac{F}{V_{\text{voi}}\cdot \rho_{\text{voi}}}. \end{array}$$

Inserting this definition into (10) yields

(12) $$\begin{array}{c} \frac{m_{c,\text{voi}}\left(t\right)}{V_{\text{voi}}}=\text{CBF}\cdot \rho_{\text{voi}}\cdot \overset{+\infty }{\underset{-\infty }{\int }}c_{\text{art}}\left(\xi \right)r\left(t-\xi \right)\text{d}\xi . \end{array}$$

According to Table 1, we define the contrast agent concentration *c*_voi_(*t*) within the volume *V*_voi_ of interest as

(13) $$\begin{array}{c} c_{\text{voi}}\left(t\right)=\frac{m_{c,\text{voi}}\left(t\right)}{V_{\text{voi}}}, \end{array}$$

which finally leads to the following formulation of the indicator-dilution theory,

(14) $$\begin{array}{cc} c_{\text{voi}}\left(t\right) & =\text{CBF}\cdot \rho_{\text{voi}}\cdot \overset{+\infty }{\underset{-\infty }{\int }}c_{\text{art}}\left(\xi \right)r\left(t-\xi \right)\text{d}\xi \\ & =\text{CBF}\cdot \rho_{\text{voi}}\cdot \left(c_{\text{art}}*r\right)\left(t\right), \end{array}$$

where ∗ denotes the convolution operator as usual, see also [21, 26]. An alternative derivation of the same mathematical result is presented in [20]. A historical overview of the development of the indicator-dilution theory with numerous references to mathematical aspects can be found in [27]. Note that the solution of (14) with respect to CBF and other clinically important perfusion parameters will be discussed in Section 3.3.

From a physiological point of view, it would be more meaningful to normalize CBF by the mass of the volume *V*_voi_*. This volume *V*_voi_* contains the mass of the parenchyma (and the interstitium) only. In that case, CBF would be a local measure for the blood volume flow per mass of parenchyma (and interstitium) that actually requires blood supply for oxygen and nutrient delivery. In (11), however, the volume *V*_voi_ also contains the mass of the blood-filled capillary bed itself. Another aspect to consider is that the mean density *ρ*_voi_ of the volume, which influences the CBF value, actually depends on the (varying) mass of the contrast agent in the capillary bed. The alternative definition of CBF,

(15) $$\begin{array}{c} \text{CB}{\text{F}}^{*}=\frac{F}{V_{\text{voi}}^{*}\cdot \rho_{\text{voi}}^{*}}, \end{array}$$

would then lead to a corresponding alternative formulation of the indicator-dilution theory,

(16) $$\begin{array}{c} c_{\text{voi}}^{*}\left(t\right)=\text{CB}{\text{F}}^{*}\cdot \rho_{\text{voi}}^{*}\cdot \left(c_{\text{art}}*r\right)\left(t\right). \end{array}$$

From a practical perspective, however, it is more convenient to use the definition of CBF given by (11), see Section 4.1.

The derivation of the indicator-dilution theory in this section was focused on brain perfusion imaging. This theoretical model can be used in stroke patients if the BBB is intact—compare Section 3.1—but it is not suited for semi-permeable tumors, for example. With slight adaptations, this theoretical model can also be applied in other applications of perfusion imaging such as pulmonary perfusion imaging. See [28] for detailed discussions. A discussion of models in hepatic and renal perfusion imaging is given in [29, 30], respectively.

In the context of perfusion measurement, the term recirculation refers to the physiological phenomenon that, due to the patient's cardiac activity, the contrast agent passes through the volume under consideration multiple times. It can easily be shown, however, that there is no need to correct for recirculation when deconvolution methods are applied to determine perfusion parameters [31].

### 3.3. Computation of Perfusion Parameters Using Deconvolution

In (14), the variables *c*_art_(*t*) and *c*_voi_(*t*) can be measured and have known values whereas the values of CBF, *r*(*t*), and *ρ*_voi_ are unknown. In order to compute CBF as well as other diagnostically relevant tissue perfusion parameters, we first need to introduce an intermediate variable, the flow-scaled residue function *k*(*t*),

(17) $$\begin{array}{c} k\left(t\right)=\text{CBF}\cdot \rho_{\text{voi}}\cdot r\left(t\right), \end{array}$$

which is given in units of 1/s and can be determined directly from the measured data *c*_art_(*t*) and *c*_voi_(*t*). Using (17), (14) can be written as

(18) $$\begin{array}{c} c_{\text{voi}}\left(t\right)=\left(c_{\text{art}}*k\right)\left(t\right). \end{array}$$

Hence, *k*(*t*) can be obtained from the measured data *c*_art_(*t*) and *c*_voi_(*t*) using a deconvolution method. Since a fundamental property of the residue function *r*(*t*) is *r*(0) = max (*r*(*t*)) = 1, we may then determine CBF as

(19) $$\begin{array}{c} \text{CBF}=\frac{1}{\rho_{\text{voi}}}\cdot \max\;\left(k\left(t\right)\right). \end{array}$$

Using max (*k*(*t*)) instead of *k*(0) has particular practical advantages that will be discussed in detail in Section 4.1.

The flow-scaled residue function *k*(*t*) can further be used to determine the MTT parameter of the tissue volume under consideration. From (2), it follows that, for *t* > 0, we have

(20) $$\begin{array}{c} \frac{\text{d}r\left(t\right)}{\text{d}t}=-h\left(t\right). \end{array}$$

Equation (1) can thus be rewritten, and then using integration by parts and (17) and (19), we obtain

(21) $$\begin{array}{cc} \text{MTT} & =\overset{\infty }{\underset{0}{\int }}\tau \;\left(-\frac{\text{d}r\left(\tau \right)}{\text{d}\tau }\right)\text{d}\tau \\ & =\overset{\infty }{\underset{0}{\int }}r\left(\tau \right)\text{d}\tau -\underset{\xi \to \infty }{\lim\;}\left({\tau r\left(\tau \right)|}_{0}^{\xi }\right)\\ & =\overset{\infty }{\underset{0}{\int }}r\left(\tau \right)\text{d}\tau \\ & =\frac{1}{\max\;\left(k\left(\tau \right)\right)}\cdot \overset{\infty }{\underset{0}{\int }}k\left(\tau \right)\text{d}\tau . \end{array}$$

Note that we have assumed that there is a constant *T* > 0 such that *r*(*t*) = 0 for *t* > *T*. This assumption ensures that

(22) $$\begin{array}{c} \underset{\xi \to \infty }{\lim\;}\left({\tau r\left(\tau \right)|}_{0}^{\xi }\right)=\underset{\xi \to \infty }{\lim\;}\left(\xi r\left(\xi \right)\right)=0. \end{array}$$

The cerebral blood volume (CBV) corresponding to the tissue volume *V*_voi_ represents another diagnostically relevant perfusion parameter and is defined as

(23) $$\begin{array}{c} \text{CBV}=\frac{V_{\text{cap}}}{\rho_{\text{voi}}\cdot V_{\text{voi}}}. \end{array}$$

It quantifies the blood volume normalized by the mass of *V*_voi_ and is typically measured in units of mL/100 g. The quantity CBV can be computed from the parameters CBF and MTT using the central volume theorem [22, 26], according to which

(24) $$\begin{array}{c} \text{CBF}=\frac{\text{CBV}}{\text{MTT}} \end{array}$$

holds for the perfused volume of interest. Interestingly, this theorem has been recognized for a long time and is already found in a historical publication from 1893 [32]. It states that the perfusion parameters CBV and CBF corresponding to the volume *V*_voi_ of interest are related by the respective temporal parameter MTT that quantifies the mean time that a blood cell needs to pass through its capillary bed. With (19) and (21), it follows from (24) that

(25) $$\begin{array}{c} \text{CBV}=\text{MTT}\cdot \text{CBF}=\frac{1}{\rho_{\text{voi}}}\cdot \overset{\infty }{\underset{0}{\int }}k\left(\tau \right)\text{d}\tau , \end{array}$$

which demonstrates that the CBV parameter can be derived from the flow-scaled residue function *k*(*t*) as well.

A healthy human brain exhibits a CBV of about 4 mL/100 g for grey matter and a CBV of about 2 mL/100 g for white matter [8].

Note that the definition of CBV that corresponds to the alternative definition of CBF in [16] is

(26) $$\begin{array}{c} \text{CB}{\text{V}}^{*}=\frac{V_{\text{cap}}}{\rho_{\text{voi}}^{*}\cdot V_{\text{voi}}^{*}}. \end{array}$$

Accordingly, this alternative definition relates the blood volume to the mass of the parenchyma (and the interstitium) only and explicitly omits the mass of the capillary bed itself.

Furthermore, there are references in the literature that suggest measuring the blood volume in units of mL/mL. This alternative dimensionless quantity may therefore be considered as a measure of blood (or vascular) volume fraction. When relating the volume *V*_cap_ of the capillary bed to the entire volume *V*_voi_ of interest, a typical average ratio of about 4% will result for the human brain. We refer to [33] for both technical and clinical details.

### 3.4. Overview of Nondeconvolution-Based Methods for Perfusion Imaging

For the sake of completeness, this section will briefly cover two alternative approaches for CBV and CBF estimation that are practical and relevant, and that do not involve deconvolution operations. Nondeconvolution-based methods for estimating perfusion parameters are also referred to as direct measurement-based approaches [26].

Firstly, there is an alternative method to compute the blood volume of the tissue volume under consideration [1]. This approach assumes that the average contrast agent concentration *c*_voi_(*t*) in the tissue volume can be related to the average contrast agent concentration *c*_cap_(*t*) in the capillary bed by

(27) $$\begin{array}{c} c_{\text{voi}}\left(t\right)=\left(\rho_{\text{voi}}\cdot \text{CBV}\right)\cdot c_{\text{cap}}\left(t\right). \end{array}$$

According to the principle of conservation of mass, it follows that

(28) $$\begin{array}{c} m_{c,\text{tot}}=F\overset{\infty }{\underset{0}{\int }}c_{\text{art}}\left(\tau \right)\text{d}\tau =F\overset{\infty }{\underset{0}{\int }}c_{\text{cap}}\left(\tau \right)\text{d}\tau =F\overset{\infty }{\underset{0}{\int }}c_{\text{ven}}\left(\tau \right)\text{d}\tau , \end{array}$$

where *m*_*c*,tot_ is the total mass of contrast agent that has passed through the volume of interest. This results in an alternative expression for CBV,

(29) $$\begin{array}{c} \text{CBV}=\frac{1}{\rho_{\text{voi}}}\cdot \frac{\int_{0}^{\infty }c_{\text{voi}}\left(\tau \right)\text{d}\tau }{\int_{0}^{\infty }c_{\text{art}}\left(\tau \right)\text{d}\tau }=\frac{1}{\rho_{\text{voi}}}\cdot \frac{\int_{0}^{\infty }c_{\text{voi}}\left(\tau \right)\text{d}\tau }{\int_{0}^{\infty }c_{\text{ven}}\left(\tau \right)\text{d}\tau }. \end{array}$$

Hence, assuming a suitable correction for contrast agent recirculation [1, 34], CBV can be estimated from the integrals of either *c*_voi_(*t*) and *c*_art_(*t*) or *c*_voi_(*t*) and *c*_ven_(*t*) over time. See [21] for details and further references with a particular focus on MR perfusion measurements.

It is argued in [34] that, particularly for the case of CT perfusion imaging of the brain, a physiologically reasonable approximation to (29) is given by

(30) $$\begin{array}{c} \text{CBV}=\frac{{\left[c_{\text{voi}}(t)\right]}_{\max\;}}{{\left[c_{\text{ven}}(t)\right]}_{\max\;}}, \end{array}$$

which avoids the computation of the integrals over time and only requires the maximum values of *c*_voi_(*t*) and *c*_ven_(*t*).

Secondly, there is a nondeconvolution-based approach to estimate the blood flow of the tissue volume under consideration; the maximum slope method [22, 34]. The derivation of this method is based on (4) and further assumes for simplicity's sake that there is no venous outflow from the tissue volume under consideration during the time of observation; that is,

(31) $$\begin{array}{c} m_{c,\text{voi}}\left(t\right)=m_{c,\text{voi},\text{in}}\left(t\right)=F\cdot \overset{t}{\underset{0}{\int }}c_{\text{art}}\left(\tau \right)\text{d}\tau . \end{array}$$

Recalling the CBF definition—compare (11)—and that *m*_*c*,voi_(*t*) = *c*_voi_(*t*) · *V*_voi_, we obtain

(32) $$\begin{array}{c} c_{\text{voi}}\left(t\right)=\rho_{\text{voi}}\cdot \text{CBF}\cdot \overset{t}{\underset{0}{\int }}c_{\text{art}}\left(\tau \right)\text{d}\tau . \end{array}$$

**Figure 5:**
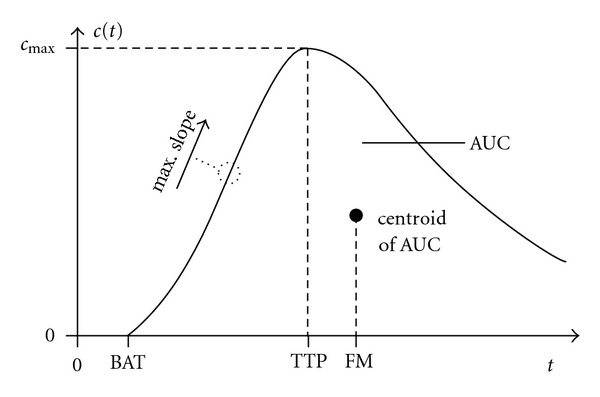
Perfusion parameters that are measured directly using the time-concentration curve. See Sections 3.5 and 4.1 for explanations (BAT: bolus arrival time, TTP: time-to-peak, FM: first moment, AUC: area under the curve).

Taking the derivative of (32) yields

(33) $$\begin{array}{c} \frac{\text{d}c_{\text{voi}}\left(t\right)}{\text{d}t}=\rho_{\text{voi}}\cdot \text{CBF}\cdot c_{\text{art}}\left(t\right), \end{array}$$

and since (33) must hold for all *t*, the blood flow is given by

(34) $$\begin{array}{c} {\left[\frac{\text{d}c_{\text{voi}}(t)}{\text{d}t}\right]}_{\max\;}=\rho_{\text{voi}}\cdot \text{CBF}\cdot {\left[c_{\text{art}}(t)\right]}_{\max\;}, \end{array}$$

which means that CBF can be estimated by dividing the maximum slope of the tissue time-concentration curve *c*_voi_(*t*), shown as an example in Figure 5, by the maximum value of the contrast agent concentration *c*_art_(*t*) in the feeding artery.

An advantage of the maximum slope method is the shorter overall acquisition time. As a downside, however, it requires a faster contrast agent bolus injection rate in order to approximately fulfill the no-venous-outflow condition.

A more comprehensive discussion of the maximum slope method and a comparison with the deconvolution method is presented in [35]. According to [35], the clinical results based on these two approaches are generally of comparably high quality in CT imaging applications. However, in cases with insufficient data quality (e.g., in terms of noise, contrast agent concentration, bolus shape), deconvolution-based methods may lead to superior results. Moreover, violation of the aforementioned no-venous-outflow condition may yield incorrect perfusion estimates when the maximum slope method is employed. This can happen for penumbral regions of the brain which characterize the tissue at risk after an ischemic stroke.

### 3.5. Additional Perfusion Parameters

Besides the aforementioned quantities CBV, CBF, and MTT, there are additional perfusion parameters such as the time-to-peak (TTP) of the time-concentration curve, the maximum contrast agent concentration *c*_max_, as well as the first moment (FM) of the time-concentration curve, for example. The first moment can be computed by projecting the centroid of the area under the curve (AUC) of the time-concentration curve onto the time axis.

Figure 5 illustrates the quantities *c*_max_, TTP, and FM. The remaining parameter bolus arrival time (BAT) will be explained in Section 4.1. In practical measurements, the time point *t* = 0 represents the start of the scanning. A detailed description and analysis of these additional quantities, however, is beyond the scope of this paper. A comparison of several perfusion parameters and their clinical impact on the treatment of stroke patients is given in [36].

In summary, Table 2 covers the most common diagnostically relevant perfusion parameters and shows how they can be determined employing deconvolution-based and nondeconvolution-based methods. In principle, the central volume theorem—compare (24)—may also be used to numerically estimate the MTT from the parameters CBF and CBV when the latter have been computed using nondeconvolution-based algorithms. However, the authors are not aware of any reference that describes the application of this approach in clinical practice.

## 4. Practical Implementation

This section is devoted to the practical computer implementation of algorithms for perfusion image analysis. First, we will discuss the necessary adaptations of the theoretical model from Section 3 that are needed for its application to data from real CT and MR scanners. Afterwards, we will describe commonly used algebraic deconvolution methods and also give an overview of alternative approaches. We will motivate the need for suitable regularization and discuss the influence of the regularization parameter on the resulting perfusion estimates. For the sake of completeness, we will also address techniques for the pre-processing of the acquired perfusion data.

### 4.1. Adaptations of the Model of Microcirculation

In Section 3.1, we presented a model of microcirculation at the tissue level. We have assumed that we can measure the average contrast agent concentration *c*_voi_(*t*) corresponding to a volume *V*_voi_ under consideration which is supplied by one single capillary bed only. Furthermore, we have supposed that we can measure the contrast agent concentration *c*_art_(*t*) locally at the arterial inlet into the capillary bed. However, real CT and MR scanners are characterized by limited spatial (and contrast) resolution and, in reality, one cannot rely on these two aforementioned assumptions. We will thus introduce two major adaptations of the physiological model which are necessary once it is to be applied to data from real scanners.

First, during a standard CT and MR perfusion exam, a volume of interest is scanned and the data is reconstructed on a grid of regularly spaced voxels. In the object domain, each voxel volume *V*_vox_ (*V*_vox_ ≫ *V*_voi_) contains numerous capillary beds as well as arterioles and venules that supply and drain these capillary beds, respectively. For the particular case when the volume *V*_vox_ is located completely within a larger artery or vein, there are of course no capillary beds located within *V*_vox_.

The measured signal (X-ray attenuation or MR relaxation rate) in a voxel is thus a combination of the signals from both the capillary beds as well as the arterial and venous vessels [37]. The perfusion parameters that are computed from the voxel's time-concentration curve are therefore not true parameters of the capillary perfusion. If no larger artery or vein is located inside the volume *V*_vox_, we may adapt the model introduced in Section 3.1 as follows: the measured time-concentration curve *c*_voi_(*t*) refers to the average perfusion from the arterioles through the capillary beds to the venules found in *V*_vox_.

The second adaptation of the model concerns the measurement of *c*_art_(*t*). In reality, it is not possible to locally measure the concentration at the arterial inlet into the volume *V*_vox_. Instead, it is common practice that a global arterial input function (AIF) is chosen in a large arterial vessel. In brain perfusion imaging, for example, the anterior cerebral artery is often selected [38].

This approach leads to a traveling time of the contrast agent bolus from where the AIF is measured to the location of the tissue volume where *c*_voi_(*t*) is measured. We will refer to this traveling time as the bolus delay. Another physical effect that needs to be taken into consideration is bolus dispersion [39]. It appears as a widening of the shape of the bolus that is caused during the flow from the remote AIF location to the measurement site of *c*_voi_(*t*).

The bolus delay has two implications. First, the curve *c*_voi_(*t*) does not start to rise at the same time point as *c*_art_(*t*) starts to rise. The difference between these two time points can be defined as the bolus arrival time (BAT), which may be considered as an additional perfusion parameter [40]. Alternatively, the BAT can be defined as the time interval between the start of the scanning and the time when *c*_voi_(*t*) begins to rise, see Figure 5. The results obtained with this alternative definition differ from the results obtained with the first definition by a constant value only.

Second, the flow-scaled residue function *k*(*t*) is equal to 0 from *t* = 0 to *t* = BAT. In addition, due to the bolus dispersion, *k*(*t*) will not rise instantaneously to its maximum at *t* = BAT, but it will have a finite rise time. The time-to-maximum (TMAX) of the flow-scaled residue function, defined as

(35) $$\begin{array}{c} \text{TMAX}=\underset{t}{\arg\;\max\;}\left(k\left(t\right)\right), \end{array}$$

has also been suggested as an additional perfusion parameter [41, 42]. Since the function *k*(*t*) can be 0 at *t* = 0 (due to bolus delay), it is reasonable and recommended to estimate CBF as the maximum of *k*(*t*)—compare (19)—and not as the value of *k*(*t*) at time *t* = 0.

Bolus delay and dispersion may lead to an underestimation of CBF [39]. In order to correct for bolus delay and dispersion several methods have been proposed [43, 44]. The use of local arterial input functions could also reduce the effect of bolus dispersion, see Section 4.5.6. On the other hand, new perfusion parameters (BAT, TMAX) are motivated by these two effects and can be defined accordingly. They represent perfusion characteristics related to the flow of the contrast agent bolus from the selected feeding artery to the respective tissue site, see again Figure 5.

### 4.2. Deconvolution Using Algebraic Methods

**Figure 6:**
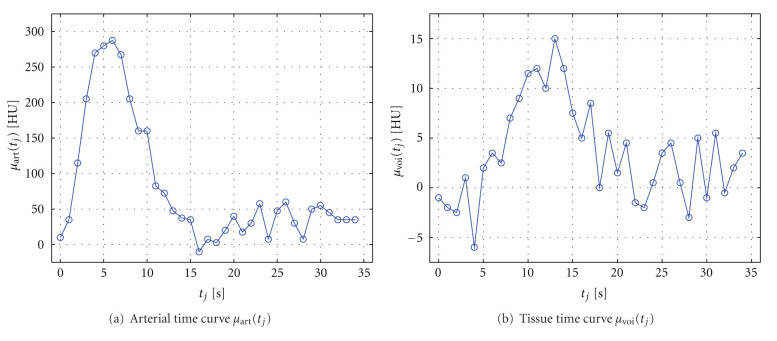
Examples of measured time-attenuation curves in perfusion CT in (a) an arterial vessel and (b) in tissue. The time curves have been pre-processed by baseline subtraction and removal of the baseline time frames. The example data is measured at *N* = 35 discrete time points.

In this section, we will discuss the robust numerical solution of the main equation of the indicator-dilution theory—(18)—by means of algebraic deconvolution methods. An overview of further deconvolution methods will then be given in Section 4.3. We will introduce the discretization of (18) and show that its solution without regularization leads to nonphysiological results. We will explain and motivate suitable regularization approaches by a singular value decomposition-based analysis. To illustrate the mathematical concepts, we will provide examples using the time-attenuation curves *μ*_art_(*t*_*j*_) and *μ*_voi_(*t*_*j*_) shown in Figure 6 that were extracted from a real perfusion CT scan.

We assume that the measured time-attenuation curves can be converted to time-concentration curves using a constant of proportionality of 1 g/mL/HU. Details about the conversion, also discussing perfusion MR data, will be explained in Section 4.5.4.

In practice, the time-concentration curves *c*_art_(*t*) and *c*_voi_(*t*) are sampled at discrete time points. We denote these time points as *t*_*j*_ = (*j* − 1) · Δ*t* with *j* = 1,…, *N*. A typical value of the sampling period Δ*t* is 1 s, for example. We can discretize (18) as

(36) $$\begin{array}{c} c_{\text{voi}}\left(t_{j}\right)=\overset{\infty }{\underset{0}{\int }}c_{\text{art}}\left(\tau \right)k\left(t_{j}-\tau \right)\text{d}\tau \approx \Delta t\overset{N}{\underset{i=1}{\sum }}c_{\text{art}}\left(t_{i}\right)k\left(t_{j-i+1}\right), \end{array}$$

see [45]. We assume that the values of *c*_art_(*t*) can be neglected for *t* > *N*Δ*t*. Since *k*(*t*) = 0 for *t* < 0, the end summation index could also be set to *j* instead of *N*. By rewriting this expression using matrix-vector notation, we obtain

(37) $$\begin{array}{cc} & \Delta t\cdot \left(\begin{array}{cccc} c_{\text{art}}\left(t_{1}\right) & 0 & \cdots & 0\\ c_{\text{art}}\left(t_{2}\right) & c_{\text{art}}\left(t_{1}\right) & \cdots & 0\\ \vdots & \vdots & \ddots & \vdots \\ c_{\text{art}}\left(t_{N}\right) & c_{\text{art}}\left(t_{N-1}\right) & \cdots & c_{\text{art}}\left(t_{1}\right) \end{array}\right)\;\;\left(\begin{array}{c} k\left(t_{1}\right)\\ k\left(t_{2}\right)\\ \vdots \\ k\left(t_{N}\right) \end{array}\right)\\ & \;\;\;=\left(\begin{array}{c} c_{\text{voi}}\left(t_{1}\right)\\ c_{\text{voi}}\left(t_{2}\right)\\ \vdots \\ c_{\text{voi}}\left(t_{N}\right) \end{array}\right), \end{array}$$

or shortly

(38) $$\begin{array}{c} Ak=c, \end{array}$$

where Δ*t* and *c*_art_(*t*_*j*_) are contained in the matrix **A** ∈ ℝ^*N*×*N*^, and *k*(*t*_*j*_) and *c*_voi_(*t*_*j*_) represent the entries of the vectors **k** ∈ ℝ^*N*^ and **c** ∈ ℝ^*N*^, respectively. Different ways to discretize (18) are investigated in [46]. For example, it was suggested in [47, 48] to use a discretization method with a block-circulant matrix **A** in order to reduce the influence of the bolus delay. See the appendix for details.

A standard approach to solve (37) for **k** is to use the singular value decomposition (SVD) of **A**. For a matrix **A** ∈ ℝ^*N*×*N*^ with *r* = rank (**A**) linearly independent rows and columns, it is defined as

(39) $$\begin{array}{c} A=U\text{Σ}V^{\text{T}}=\overset{r}{\underset{i=1}{\sum }}u_{i}\sigma_{i}v_{i}^{\text{T}}, \end{array}$$

where **U** = [**u**_1_,…, **u**_*r*_] and **V** = [**v**_1_,…, **v**_*r*_] are unique orthogonal matrices composed of the left and right singular vectors **u**_*i*_ and **v**_*i*_, respectively [49]. The number of rows and columns in **A** that only contain zeros is determined by the number *N*_lz_ of leading zeros in the series *c*_art_(*t*_*j*_), *j* = 1,…, *N*. Therefore, **A** has rank *r* ≤ *N* − *N*_lz_. After the subtraction of the baseline, it may happen that the first entry *c*_art_(*t*_1_) is zero, see Section 4.5.4, and that **A** thus becomes rank-deficient. The diagonal matrix *𝚺* = diag (*σ*_1_,…, *σ*_*r*_) contains the singular values *σ*_*i*_ in nonincreasing order *σ*_1_ ≥ *σ*_2_ ≥ ⋯≥*σ*_*r*_ > 0. The least-squares solution **k**_ls_ of (38) using the SVD of **A** is given by

(40) $$\begin{array}{c} k_{\text{ls}}=\overset{r}{\underset{i=1}{\sum }}\frac{u_{i}^{\text{T}}c}{\sigma_{i}}v_{i}, \end{array}$$

see again [49]. Note that the unique vector **k**_l*s*_ is referred to as least-squares solution since determining it from (40) is equivalent to minimizing the squared Euclidean residual norm of the linear systems given by (37) and (38); that is,

(41) $$\begin{array}{c} k_{\text{ls}}=\underset{k\in {\mathbb{R}}^{N}}{\arg\;\min\;}\left({||Ak-c||}_{2}^{2}\right). \end{array}$$

However, the least-square solution **k**_ls_ does not represent a suitable solution of (38) if the matrix **A** is ill-conditioned. It can be shown that a matrix **A** with a structure as shown in (37) or (A.3), also known as a Toeplitz matrix, is in fact ill conditioned [50, 51]. In that case, a small change in **c** (e.g., due to projection noise) can cause a large change in **k**_ls_. The rate at which a change in **c** influences the solution **k**_ls_ is roughly proportional to the condition number of **A**, defined as *σ*_1_/*σ*_*r*_ [49].

**Figure 7:**
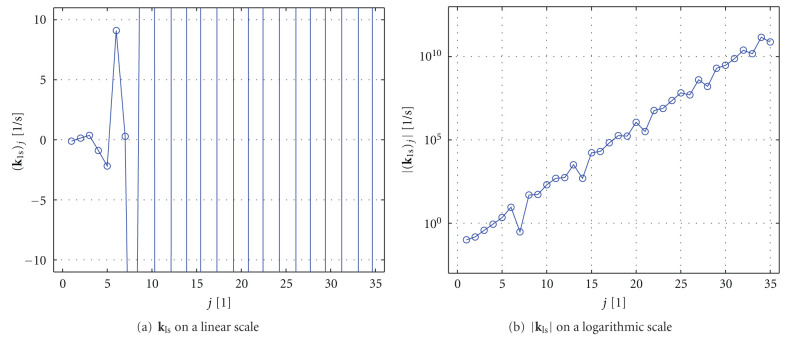
Least-squares solution vector **k**_ls_ of (38) using the example data from Figure 6. (**k**_ls_)_*j*_ denotes the *j*th entry of the vector **k**_ls_. The plot shown in (a) illustrates the strong oscillations of **k**_ls_. The plot given in (b) shows the amplitude |**k**_ls_| of this function on a logarithmic scale.

As an example, Figure 7 shows the solution **k**_ls_ of the example data from Figure 6. The solution is strongly oscillating and even has a rising amplitude. It is obvious that this solution has nothing in common with the real physiological behavior of the flow-scaled residue function.

**Figure 8:**
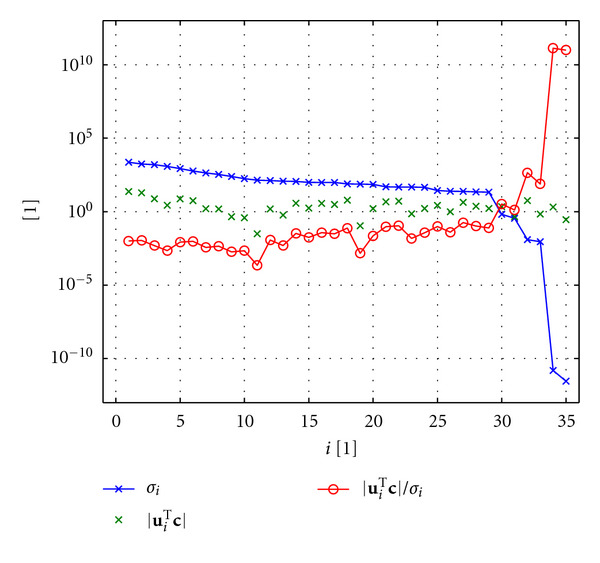
SVD analysis of the matrix **A** constructed from the example data shown in Figure 6. The plot displays the absolute values of the weighting factors (**u**_*i*_^*T*^**c**)/*σ*_*i*_ and of their individual components |**u**_*i*_^T^**c**| and *σ*_*i*_ on a logarithmic scale.

In order to get a better understanding of why **k**_ls_ is not a meaningful solution and to motivate the regularization approach, we will investigate the individual terms of (40). We use the data shown in Figure 6 to obtain **A** and **c**.  Figure 8 represents a plot of the absolute values of the expressions (**u**_*i*_^T^**c**)/*σ*_*i*_ that occur in (40). These factors weight the right singular vectors **v**_*i*_ of **A**.

It is known from numerical analysis that the discrete Picard condition represents a means to analyze discrete ill-conditioned problems [50, 51]. This condition is violated, if the expressions **u**_*i*_^T^**c** do not decay faster, on average, than the singular values *σ*_*i*_ until a threshold value is reached where the singular values level off. The reader is referred to [51] for a more detailed explanation of the discrete Picard condition and its relation to the Picard condition from which it is derived. A usual reason for the violation of the discrete Picard condition is noise in the measured data that the matrix **A** is based on. We can see that the discrete Picard condition is actually violated in the example shown in Figure 8 [52]. Consequently, the absolute values of the ratios (**u**_*i*_^T^**c**)/*σ*_*i*_—which represent the weighting factors of the right singular vectors **v**_*i*_—become very large.

To obtain a numerically stable result, a filter is used for regularization. The filter should suppress the influences of small singular values *σ*_*i*_ or, equivalently, the influences of high absolute values of the weighting factors (**u**_*i*_^T^**c**)/*σ*_*i*_. The regularized solution **k**_*λ*_, where *λ* is a regularization parameter, is given by

(42) $$\begin{array}{c} k_{\lambda }=\overset{r}{\underset{i=1}{\sum }}\left(f_{\lambda ,i}\frac{u_{i}^{\text{T}}c}{\sigma_{i}}\right)v_{i}. \end{array}$$

We will focus on two common definitions of the filter factors *f*_*λ*,*i*_. Firstly, the filter factors *f*_*λ*,*i*_^  (tsvd)^ correspond to the truncated singular value decomposition (TSVD) approach and are defined with a sharp threshold at *λ*,

(43) $$\begin{array}{c} f_{\lambda ,i}^{\;(\text{tsvd})}=\{\begin{array}{cc} 0, & \text{for}\;\sigma_{i}<\lambda ,\\ 1, & \text{for}\;\sigma_{i}\geq \lambda . \end{array} \end{array}$$

Secondly, the filter factors *f*_*λ*,*i*_^  (tikh)^ are based on the Tikhonov regularization approach and characterized by a smooth weighting function centered around *λ*,

(44) $$\begin{array}{c} f_{\lambda ,i}^{\;(\text{tikh})}=\frac{\sigma_{i}^{2}}{\sigma_{i}^{2}+\lambda^{2}}. \end{array}$$

The (absolute) regularization parameter *λ* is usually computed relative to the maximum singular value *σ*_1_, that is,

(45) $$\begin{array}{c} \lambda =\lambda_{\mathrm{rel}\;}\sigma_{1}. \end{array}$$

The relative regularization parameter *λ*_*rel* _ is supposed to lie in the interval between 0 and 1.

**Figure 9:**
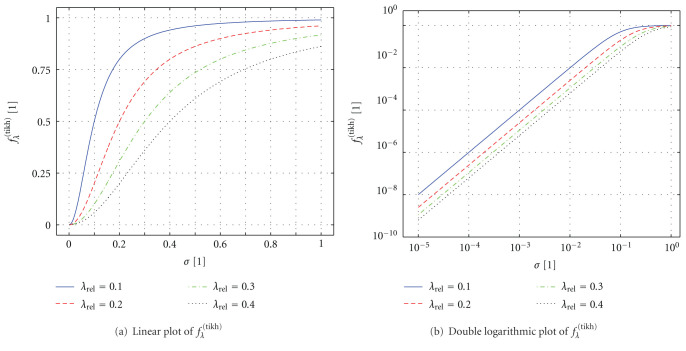
(a) Linear and (b) double logarithmic plot of the Tikhonov filter factor *f*_*λ*_^(tikh)^ as a function of the singular value *σ* ∈ [10^−5^, 1].

In order to illustrate the Tikhonov filter factors, Figure 9 shows a plot of the function *f*_*λ*_^  (tikh)^ = *σ*^2^/(*σ*^2^ + *λ*^2^) which is—unlike (44)—defined for a continuous range of *σ*. For determining *f*_*λ*_^  (tikh)^, we assumed *σ*_1_ = 1. It can be seen that, for increasing *λ* (i.e., stronger regularization), the values of *f*_*λ*_^  (tikh)^ decrease for all *σ*.

Interestingly, the solution **k**_*λ*_^(tikh)^ of (38) using the filter factors *f*_*λ*,*i*_^  (tikh)^ is equivalent to minimizing the weighted sum of the squared Euclidean residual norm ||**Ak** − **c**||_2_^2^ and the squared Euclidean solution norm ||**k**||_2_^2^; that is,

(46) $$\begin{array}{c} k_{\lambda }^{\left(\text{tikh}\right)}=\underset{k\in {\mathbb{R}}^{N}}{\arg\;\min\;}\left({||Ak-c||}_{2}^{2}+\lambda^{2}{||k||}_{2}^{2}\right). \end{array}$$

**Figure 10:**
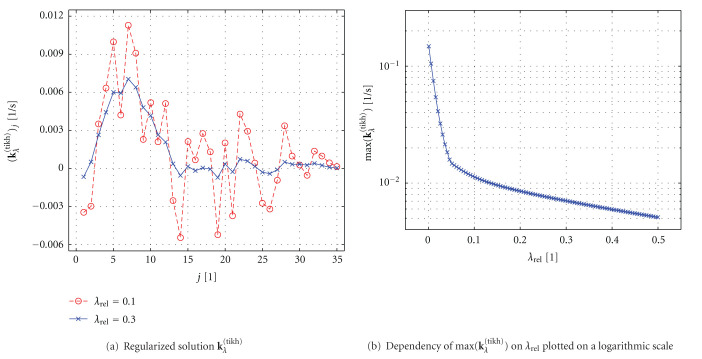
Deconvolution with Tikhonov regularization: (a) Regularized solution **k**_*λ*_^(tikh)^ for two different regularization parameters *λ*_*rel* _ and (b) maximum of **k**_*λ*_^(tikh)^ as a function of *λ*_*rel* _. (**k**_*λ*_^(tikh)^)_*j*_ denotes the *j*th entry of the vector **k**_*λ*_^(tikh)^.

Figure 10(a) shows the solution **k**_*λ*_^(tikh)^ computed for two different regularization parameters. The solution for *λ*_*rel* _ = 0.1 still shows some nonphysiological oscillations. However, the solution for *λ*_*rel* _ = 0.3 can in fact be interpreted as a flow-scaled residue function in the presence of bolus delay and dispersion, compare Section 4.1.  Figure 10(b) illustrates a plot of max (**k**_*λ*_^(tikh)^), which is proportional to CBF (see Section 3.3 and Table 2), as a function of *λ*_*rel* _. Apparently, CBF depends on the choice of regularization parameter. Choosing an optimal regularization parameter that will lead to physiologically reasonable estimates will be discussed in Section 4.4.

### 4.3. Alternative Deconvolution Approaches

 The algebraic deconvolution approach from Section 4.2 is very commonly applied to analyze perfusion data. Yet, deconvolution problems arise in many other applications, and numerous alternative algorithms to solve these problems have been developed [53]. In this section, we provide a brief overview of alternative deconvolution approaches that have also been applied to perfusion data.

The Fourier transform represents a standard method to solve deconvolution problems [54], and it has also been evaluated to analyze perfusion data [45, 55–57]. Interestingly, the Fourier transform-based deconvolution approach is mathematically equivalent to the SVD-based deconvolution approach with a block-circulant matrix **A**, compare the appendix [47, 58–60]. However, results obtained with SVD-based and FT-based deconvolution can be different because the chosen regularization approaches for these two methods are usually not equivalent. The regularization in the context of the Fourier-based deconvolution approach can be implemented by means of a modified Wiener filter [55], for example. The reader is referred to [60, 61] for a detailed analysis of the equivalence of SVD-based and Fourier-based regularization approaches.

In contrast to the model-independent deconvolution approaches also model-dependent approaches exist. Model-dependent approaches assume a certain shape of the residue function. For example, in [45, 62] a decaying exponential function was used which makes the deconvolution more stable since it reduces the degrees of freedom of the residue function [45]. However, if the underlaying residue function is different from the model the perfusion parameters may not be estimated correctly.

Deconvolution using orthogonal polynomials was investigated in [63]. An iterative deconvolution algorithm based on maximum likelihood expectation maximization (ML-EM) algorithm was proposed in [64]. An approach using Gaussian processes was evaluated in [65]. The deconvolution algorithm in [66] uses a nonlinear stochastic regularization method.

A comprehensive comparison of all available deconvolution methods has not been carried out yet. The SVD-based deconvolution approach, which is available in several software packages [67–69], is comparably simple to implement and can be considered as the current standard method in perfusion image analysis.

### 4.4. Determination of the Regularization Parameter

Figure 10(b) demonstrated that (the maximum of) the solution **k**_*λ*_^(tikh)^ depends on the regularization parameter *λ*_*rel* _. Consequently, the computed perfusion values—which can be derived from **k**_*λ*_^(tikh)^ according to Table 2—vary for different *λ*_*rel* _. As an example, the CBF value will be underestimated systematically for large *λ*_*rel* _.

Therefore, an optimal choice of *λ*_*rel* _ is crucial. A simple approach is to empirically determine a fixed value *λ*_*rel* _. This approach is often used in practice, and a typical value in brain perfusion CT is, for example, *λ*_*rel* _ = 0.2 [68]. However, there exist more sophisticated approaches as well to determine the values *λ*_*rel* _ independently for each voxel position [70]. Since the required amount of regularization depends roughly on the signal-to-noise ratio (SNR), these approaches can be more flexible when the SNR is spatially variant.

In [45, 47, 48], an oscillation index (OI) was defined to determine the intensity of oscillations of the flow-scaled residue function. The regularization can then be varied until the OI value falls below a certain threshold.

The L-curve criterion represents a model-independent method to determine *λ* (and *λ*_*rel* _) [31, 71, 72]. The L-curve is defined by a double logarithmic plot of the squared Euclidean norm ||**k**_*λ*_||_2_^2^ of the solution versus the squared Euclidean norm ||**Ak**_*λ*_ − **c**||_2_^2^ of the residual for a range of different *λ* values. The optimal regularization parameter *λ*_opt_ can be found at the location of the characteristic corner point of the L-curve.

Another method to determine an appropriate regularization parameter is generalized cross-validation as described in [50, 73]. An implementation of the L-curve method and the generalized cross-validation can be found in [52].

Furthermore, a parameter estimation method that uses a priori knowledge of the behavior of the residue function was proposed in [74].

Kudo et al. [68] reported that two manufacturers applied a fixed threshold value *λ*_*rel* _ in their perfusion analysis software. Unfortunately, the clinical use of methods with adaptive threshold values is rarely described in the current literature.

### 4.5. Perfusion Data Preprocessing

This section gives an overview of pre-processing techniques that can be applied in order to enhance the quality of the estimated perfusion parameters. Pre-processing occurs prior to the deconvolution step which may be implemented as described in Section 4.2.

A simple, yet mandatory, pre-processing step consists of the conversion to contrast agent concentration values, see Section 4.5.4. Further pre-processing steps are used to enhance the image quality (e.g., noise reduction) and to correct for artifacts (e.g., motion correction, partial volume correction) and specific properties of the blood (e.g., correction of differences in hematocrit). There are also pre-processing steps that can optimize the analysis of the perfusion value maps (e.g., segmentation of certain anatomic structures) and the application workflow (e.g., automated AIF estimation).

The order of the pre-processing steps presented in this section can act as a guideline for their practical implementation. However, a different ordering can of course be reasonable as well. Finally, this overview cannot include all details regarding suitable pre-processing steps. The reader is referred to the available literature for in-depth discussions.

#### 4.5.1. Motion Correction

 Patient motion (e.g., due to head movement or breathing) can result in a sudden change of the attenuation values at the fixed (stationary) voxel positions. Since this change in the attenuation value is caused by motion and not by contrast agent flow, the computed perfusion values can be severely biased. A practical approach for motion correction is to register all time frames of the reconstructed data set onto the first time frame [75]. A 3D registration should be used because it can also correct motion that occurs perpendicular to the orientation of the reconstructed slices. For a brain perfusion scan, a rigid registration may be sufficient. Conversely, in abdominal perfusion imaging, a non-rigid registration may be better suited to compensate for the deformations due to breathing.

As an alternative to registration, use of groupwise motion correction based on an optimization of a global cost function has been suggested [76]. There are also several approaches for motion correction in fMRI data [77]. These approaches may be used for perfusion MR data as well since both types of data typically consist of *T*2*-weighted EPI images [78]. However, the dynamic signal changes are relatively higher in DSC-MR data when compared to fMRI data [78]—in particular during the contrast agent bolus passage—which must be taken into account when adapting fMRI-based motion correction algorithms to DSC-MR data.

A related issue is streak artifact in reconstructed perfusion CT images that are caused by patient motion that occurs while the projection data corresponding to a single time frame is acquired. In perfusion MR images, ghosting artifacts can arise if the patient moves during the data acquisition. These kinds of artifact cannot be corrected by inter-frame motion correction. Instead, dedicated reconstruction algorithms would be required. As a practical alternative, time frames that exhibit severe reconstruction artifacts may simply be removed from the data set (i.e., from the series of successive time frames), which corresponds to the elimination of invalid sampling points of the voxel-specific time-concentration curves.

#### 4.5.2. Noise Reduction

 In the course of a perfusion exam, the measured signal in tissue that is caused by the contrast agent flow can be very low. For the case of perfusion CT, for example, tissue enhancements of less than 10 HU are measured. Hence, noise in the reconstructed images can be of a similar order of magnitude as the signal in tissue itself. Consequently, noise reduction should be taken into consideration in order to improve the accuracy of the estimated perfusion parameters.

Noise reduction can be implemented as a spatial smoothing of the data. Using a basic approach, each time frame can be filtered independently of the other time frames, and linear isotropic filters (e.g., based on a Gaussian filter kernel) may be applied. Alternatively, anisotropic filters that preserve edges and avoid blurring of large vessels can also be employed [79].

Both linear and nonlinear filtering in the temporal dimension—that is, between successive time frames—represent further methods for noise reduction [80]. It should be noted, however, that the regularization during the deconvolution step is equivalent to linear filtering in the temporal domain.

Recently, sophisticated 4D filtering techniques have been proposed that perform filtering in both the spatial and the temporal dimension and that are optimized for perfusion data [81, 82]. Fitting of the time-concentration curves to a model function such as a gamma-variate function is also a means for noise reduction [75].

#### 4.5.3. Segmentation

 A segmentation of certain anatomic structures in the reconstructed data set can optimize the perfusion image analysis [69, 83]. For example, the time-concentration curves could then be analyzed only in regions of interest where blood flow is actually expected [84]. Other regions such as air, bone, cerebrospinal fluid (CSF), and calcifications can be neglected. A segmentation and the subsequent removal of vessels is useful in order to optimize the quantitative analysis of perfusion parameters in tissue. Such a vessel segmentation can be performed prior to the deconvolution step, but it can also be implemented as a postprocessing step as described in [85].

#### 4.5.4. Conversion to Contrast Agent Concentration

 Neither for the case of CT imaging nor for the case of MR imaging can the time-concentration curves *c*_art_(*t*) and *c*_voi_(*t*) be measured directly. Instead, the measurement is a superposition of the signal from the tissue itself and the contrast agent. Since the deconvolution approach presented in Section 4.2 expects that the functions *c*_art_(*t*_*j*_) and *c*_voi_(*t*_*j*_) only refer to the signal caused by the contrast agent, the tissue signal must be subtracted. Furthermore, the measured signal must be converted to a contrast agent concentration value.

In perfusion CT, it is assumed that the (underlying) contrast agent concentration value is proportional to the (measured) X-ray attenuation value [86, 87]. Since deconvolution is a linear operation, the constant of proportionality does not influence the computed flow-scaled residue function. It can also be seen that the additional perfusion parameters from Section 3.5 are independent of this constant. Therefore, this constant is usually set to *k*_ct_ = 1 g/mL/HU for the sake of simplicity. The baseline value *μ*_0_ can be computed as the mean of *μ*(*t*_*j*_) during the *B* acquired time frames before the contrast agent bolus arrives in the arterial input function. The conversion formula from an attenuation value *μ*(*t*_*j*_) (corresponding to a particular voxel) into the respective contrast agent concentration value *c*(*t*_*j*_) then reads as

(47) $$\begin{array}{c} c\left(t_{j}\right)=k_{\text{ct}}\left(\mu \left(t_{j+B-1}\right)-\mu_{0}\right),\\ \mu_{0}=\frac{1}{B}\;\overset{B}{\underset{i=1}{\sum }}\;\mu \left(t_{i}\right). \end{array}$$

In perfusion MR, however, the contrast agent concentration value is not proportional to the received signal *s*(*t*_*j*_) (in one voxel). Instead, it can be determined using the following formula:

(48) $$\begin{array}{c} c\left(t_{j}\right)=-\frac{k_{\text{mr}}}{\text{TE}}\ln\;\left(\frac{s\left(t_{j+B-1}\right)}{s_{0}}\right),\\ s_{0}=\frac{1}{B}\;\overset{B}{\underset{i=1}{\sum }}\;s\left(t_{i}\right), \end{array}$$

see [21]. Here, *k*_mr_ is a constant of proportionality which—with a similar argument as for *k*_ct_—can have a norm of 1 and TE is the echo time of the MR sequence. It must be noted, however, that the constant *k*_mr_ can be different for blood and tissue due to differences in *T*2* relaxivities [37, 88]. This complicates absolute quantification of cerebral perfusion as discussed in [89]. Furthermore, studies have shown that fully oxygenated blood, for example, demonstrates a nonlinear relationship between the measured difference in *T*2* relaxation rate and contrast agent concentration [90].

Note that if only one time frame is considered as the baseline (i.e., if *B* = 1), then *c*(0) = 0, and the matrix **A** defined by (37) and (38) will be rank deficient, compare Section 4.2.

#### 4.5.5. Correction of Hematocrit Differences

Hematocrit (Hct) is a value that describes the proportion of the blood that consists of red blood cells. Hct is higher in arteries than in capillaries. Consequently, the proportion of the plasma in the blood, given by the difference (1-Hct), has a higher value in capillaries than in arteries. Since the contrast agent is distributed in the plasma only, the amount of plasma has a direct influence on the measured Hounsfield value or MR relaxation rate.

If the Hct difference is not corrected, it may bias the absolute quantification of the contrast agent concentration. A constant dimensionless correction factor *κ*, derived from the known Hct values in arteries and capillaries (often set to *κ* = 0.73) has been proposed [22, 85]. The measured time-concentration curve *c*_voi_(*t*) is then multiplied with *κ* to avoid the bias due to different Hct values.

#### 4.5.6. Automated AIF Estimation

 The total time for the perfusion image analysis can be shortened and the analysis can be made user independent by an automated estimation of the arterial input function. Several methods have been proposed that detect one global AIF [91–93].

An interesting alternative approach is to estimate several local AIFs, which would be better suited to the theoretical model that was introduced in Section 3 [94–96]. Since the local arteries are often small, this approach can have several disadvantages [89]. For example, partial volume effects—compare Section 4.5.7—can be more severe when compared to choosing one global AIF in a larger vessel. Perfusion analysis using local AIFs is actually investigated in [97] and the authors state that it produced more useful CBF maps.

Besides the arterial input function *c*_art_(*t*) the venous outflow function *c*_ven_(*t*) could also be detected automatically. Knowledge about the venous outflow function could be used to automatically correct for partial volume effects which are described next.

#### 4.5.7. Correction of Partial Volume Effects in the AIF

 Due to limited spatial resolution in reconstructed perfusion CT and MR data, the AIF can suffer from partial volume effects [26]. This effect can lead to an underestimation of the AIF and consequently to incorrect perfusion values. To correct for partial volume effects in the AIF, several methods have been proposed [98–100]. Commonly, the peak concentration value within a larger venous vessel or the area under the curve of a large venous vessel is used to rescale the AIF [31].

## 5. Summary

We have presented an overview of algorithms for the estimation of the most prominent perfusion parameters from CT or MR measurements that play an essential role in the assessment of flow altering diseases such as stroke, for example. In particular, we have emphasized the class of deconvolution-based methods that result from the application of the indicator-dilution theory, which is also derived in detail. Alternative approaches that do not use a deconvolution method are addressed briefly as well. The robust numerical solution of the resulting system of linear equations represents the second major topic of this paper. We have included a detailed discussion regarding the application of the singular value decomposition method as well as the practically relevant introduction of a suitable regularization technique in order to avoid physiologically unrealistic behavior of the estimated solution. Since this paper is intended to provide an introduction both to the underlying theory and to implementation-relevant aspects, we have provided a survey of preprocessing techniques that should be considered when designing a clinically useful tool for CT or MR perfusion analysis.

The novel contribution of this paper is to present the fundamental model, the mathematical deconvolution with regularization, and the practical pre-processing steps in one place. For a thorough understanding of perfusion image analysis, knowledge of all of these aspects is important and we have elaborated several links between these topics.

## Figures and Tables

**Table 1:** Summary of parameters used to derive the indicator-dilution theory and to define clinically relevant tissue perfusion quantities.

Variable	Unit	Description
*V*_voi_	mL	Total volume under consideration
*V*_cap_	mL	Volume of the capillary bed within the volume *V*_voi_
*V*_voi_*	mL	Volume *V*_voi_ without the volume of the capillary bed, *V*_voi_* = *V*_voi_ − *V*_cap_
*ρ*_voi_	g/mL	Mean density of the volume *V*_voi_
*ρ*_voi_*	g/mL	Mean density of the volume *V*_voi_*
*m*_c,voi_(*t*)	g	Total mass of contrast agent in volume *V*_voi_
*c*_art_(*t*)	g/mL	Local contrast agent concentration at the arterial inlet, *c*_art_(*t*) = d*m*/d*V*|_*t*_, measured at the arterial inlet
*c*_ven_(*t*)	g/mL	Local contrast agent concentration at the venous outlet, *c*_ven_(*t*) = d*m*/d*V*|_*t*_, measured at the venous outlet
*c*_voi_(*t*)	g/mL	Average contrast agent concentration in the total volume *V*_voi_, *c*_voi_(*t*) = *m*_*c*,voi_(*t*)/*V*_voi_
*c*_cap_(*t*)	g/mL	Average contrast agent concentration in the capillary bed, *c*_cap_(*t*) = *m*_*c*,voi_(*t*)/*V*_cap_
*c*_voi_*(*t*)	g/mL	Average contrast agent concentration corresponding to *V*_voi_*, *c*_voi_*(*t*) = *m*_*c*,voi_(*t*)/*V*_voi_*
*F*	mL/s	Volume flow at the arterial inlet and at the venous outlet
*h*(*t*)	1/s	Probability density function of the transit times

**Table 2:** Summary of perfusion parameters and how these parameters can be estimated using deconvolution-based and nondeconvolution-based methods.

Parameter	w/Deconvolution	w/o Deconvolution
CBV	(1/*ρ*_voi_) · ∫_0_^*∞*^*k*(*τ*)d*τ*	(1/*ρ*_voi_) · ∫_0_^*∞*^*c*_voi_(*τ*)d*τ*/∫_0_^*∞*^*c*_art_(*τ*)d*τ*
CBF	(1/*ρ*_voi_) · max (*k*(*t*))	(1/*ρ*_voi_) · [d*c*_voi_(*t*)/d*t*]_max _/[*c*_art_(*t*)]_max _
MTT	∫_0_^*∞*^*k*(*τ*)d*τ*/max (*k*(*τ*))	see comment in Section 3.5
TTP	—	arg max _*t*_(*c*_voi_(*t*))
FM	—	∫_0_^*∞*^*c*_voi_(*τ*)*τ*d*τ*/∫_0_^*∞*^*c*_voi_(*τ*)d*τ*

## Appendix

The matrix **A** in (38) can be replaced by a block-circulant matrix **A**_circ_ to reduce the influence of the bolus delay, compare Section 4.1, and thus to become independent of time shifts in the tissue time-concentration curve. Several studies actually exhibited an improvement of the accuracy of the perfusion estimates when using this alternative discretization method compared to the approach given by (36) [47, 48, 101]. On the other hand, in a receiver operating characteristics analysis—concerning infarct prediction in acute stroke patients—both discretization methods led to almost equal results [36].

The elements *a*_*i*,*j*_ of **A** ∈ ℝ^*N*×*N*^—with *i* denoting the row index (*i* = 1,…, *N*) and *j* denoting the column index (*j* = 1,…, *N*) as usual—are defined as

(A.1)
$$\begin{array}{c} a_{i,j}=\{\begin{array}{cc} \Delta tc_{\text{art}}\left(t_{i-j+1}\right), & \text{for}\;j\leq i,\\ 0, & \text{for}\;j>i, \end{array} \end{array}$$

see (37). In order to assemble the block-circulant matrix **A**_circ_, the size of the time series *c*_art_(*t*_*j*_) must be increased from *N* to *M* (*M* ≥ 2*N*) using zero padding. We denote the new zero-padded time series as ${\tilde{c}}_{\text{art}}(t_{j})$. The size of *c*_voi_(*t*_*j*_) must be changed accordingly in order to retain consistency in (38).

The elements (*a*_circ_)_*i*,*j*_ of the block-circulant matrix **A**_circ_ ∈ ℝ^*M*×*M*^ can then be defined as

(A.2)
$$\begin{array}{c} {\left(a_{\text{circ}}\right)}_{i,j}=\{\begin{array}{cc} \Delta t{\tilde{c}}_{\text{art}}\left(t_{i-j+1}\right), & \text{for}\;j\leq i,\;\\ \Delta t{\tilde{c}}_{\text{art}}\left(t_{M+i-j+1}\right), & \;\text{for}\;j>i. \end{array} \end{array}$$

As an example, for *M* = 2*N*, the matrix **A**_circ_ has the following structure:

(A.3)
$$\begin{array}{c} A_{\text{circ}}=\Delta t\left(\begin{array}{cccccccc} c_{\text{art}}\left(t_{1}\right) & 0 & \cdots & 0 & 0 & c_{\text{art}}\left(t_{N}\right) & \cdots & c_{\text{art}}\left(t_{2}\right)\\ c_{\text{art}}\left(t_{2}\right) & c_{\text{art}}\left(t_{1}\right) & \cdots & 0 & 0 & 0 & \cdots & c_{\text{art}}\left(t_{3}\right)\\ \vdots & \vdots & \ddots & \vdots & \vdots & \vdots & \ddots & \vdots \\ c_{\text{art}}\left(t_{N}\right) & c_{\text{art}}\left(t_{N-1}\right) & \cdots & c_{\text{art}}\left(t_{1}\right) & 0 & 0 & \cdots & 0\\ 0 & c_{\text{art}}\left(t_{N}\right) & \cdots & c_{\text{art}}\left(t_{2}\right) & c_{\text{art}}\left(t_{1}\right) & 0 & \cdots & 0\\ 0 & 0 & \cdots & c_{\text{art}}\left(t_{3}\right) & c_{\text{art}}\left(t_{2}\right) & c_{\text{art}}\left(t_{1}\right) & \cdots & 0\\ \vdots & \vdots & \ddots & \vdots & \vdots & \vdots & \ddots & \vdots \\ 0 & 0 & \cdots & 0 & c_{\text{art}}\left(t_{N}\right) & c_{\text{art}}\left(t_{N-1}\right) & \cdots & c_{\text{art}}\left(t_{1}\right) \end{array}\right). \end{array}$$

The horizontal and vertical lines drawn in (A.3) subdivide the matrix into four quadrants. As can be seen, the matrix **A** is a submatrix of **A**_circ_, and it appears in the upper left and lower right quadrant.
